# Real-time RT-PCR for COVID-19 diagnosis: challenges and prospects

**DOI:** 10.11604/pamj.supp.2020.35.24258

**Published:** 2020-07-21

**Authors:** Waidi Folorunso Sule, Daniel Oladimeji Oluwayelu

**Affiliations:** 1 Department of Microbiology, Osun State University, Oke-Baale, Osogbo 230212, Nigeria,; 2 Department of Veterinary Microbiology, University of Ibadan, Ibadan 200284, Nigeria

**Keywords:** COVID-19, SARS-CoV-2, diagnosis, RT-qPCR, reliability

## Abstract

COVID-19 impacts global public health, economy, education, tourism/hospitality and sports; rapid and accurate testing of clinical samples dictate effective response. So far, the real-time reverse transcriptase-polymerase chain reaction (RT-qPCR) is the assay of choice for COVID-19 diagnosis considering its rapidity and accuracy in informing on active coronavirus (CoV) infection. Presently, several RT-qPCR protocols with differing sensitivity/specificity are used for performing this assay; some of them are known to have generated debatable test results to constitute challenges worthy of consideration. This review provides a critical assessment of various published works on RT-qPCR assays used for COVID-19 diagnosis with their different indicators of positivity i.e., cycle threshold (Ct) cut-off values. Knowledge of diagnostic tests for COVID-19 is still evolving and, as a prospect, underscores the need for local validation of positive-negative Ct cut-off values when establishing RT-qPCR assays for SARS-CoV-2 detection.

## Commentary

Following the outbreak of a severe acute respiratory disease caused by a newly emerged virus among humans in Wuhan city, Hubei Province, China in December, 2019, the causal agent was initially called 2019-novel coronavirus (2019-nCoV) while the associated respiratory illness was officially designated Coronavirus Disease 2019 (COVID-19). However, with the availability of the complete genome sequence of this virus, the Coronavirus Study Group (CSG) of the International Committee on Taxonomy of Viruses renamed it severe acute respiratory syndrome-coronavirus type 2 (SARS-CoV-2) based on comparative analysis with the 2002 SARS-CoV [1]. The virus is a positive-sense, single-stranded RNA virus in the genus *Betacoronavirus*, family *Coronaviridae*. As of 3 June, 2020, the number of laboratory-confirmed cases of COVID-19 reported worldwide was 6,432,370 with 385,991 deaths [2]. According to Sharfstein et al. [3], the pivot of an effective human response to this COVID-19 pandemic is early, rapid and accurate testing of clinical samples from suspected and probable cases. The diagnostic test is strategic to interrupting transmission of SARS-CoV-2 as it identifies infected persons for appropriate clinical management in isolation facilities, contact-tracing and guides provision of real-time epidemiologic/surveillance information to the public for infection prevention and control (IPC) purposes. There are two major approaches to diagnosis of COVID-19: (1) laboratory tests that detect the SARS-CoV-2 (or its RNA or protein) in clinical samples of infected persons, including nasal/nasopharyngeal swabs, sputum, bronchoalveolar lavage fluid, faeces, and saliva; (2) laboratory tests that detect evidence of host immune response to the virus [4].

The real-time reverse transcriptase-polymerase chain reaction (RT-qPCR) is the molecular-based assay used globally to detect SARS-CoV-2 RNA in clinical samples of patients manifesting COVID-19 compatible signs and symptoms (fever, fatigue, chills, dry cough, sneezing, dyspnea, myalgia, lymphopenia and radiographic findings of pneumonia) [5]. This assay has the capacity to detect and measure minute amounts of nucleic acids in different sample types from various sources (environmental or clinical). It has been described as the enabling technology *par excellence* for molecular diagnostics covering life sciences, agriculture and medicine [6]. The RT-qPCR is highly sensitive and specific (though not 100% for each), rapid and widely used for pathogen detection [6]. These attributes make it perhaps the best diagnostic test available for rapid detection of lethal pathogens with pandemic potential, such as SARS-CoV-2, in clinical samples. Several modifications of this assay have been developed by different laboratories for testing COVID-19 samples (Table 1) [7], with the World Health Organization (WHO) approving some of them for use in laboratory testing of samples in the ongoing pandemic.

**Table 1:** common RT-qPCR assays used for COVID-19 diagnosis

Institute	Target	Primer/Probe^#^	Reference
Charite	E	E_Sarbeco_F	Corman *et al*. (2020)
		E_Sarbeco_R	
		E_Sarbeco_P1	
	RdRp	RdRp_SARSr-F	
		RdRp_SARSr-R	
		RdRp_SARSr-P1	
		RdRp_SARSr-P2	
HKU	N	HKU-N-F	Chu *et al*. (2020)
		HKU-N-R	
		HKU-N-P	
	Nsp14	HKU-ORF1-F	
		HKU-ORF1-R	
		HKU-ORF1-P	
China CDC	N	CCDC-N-F	China CDC (2020)
		CCDC-N-R	
		CCDC-N-P	
	nsp10	CCDC-ORF1-F	
		CCDC-ORF1-R	
		CCDC-ORF1-P	
US CDC	N	2019-nCoV_N1-F	CDC, 2020
		2019-nCoV_N1-R	
		2019-nCoV_N1-P	
	N	2019-nCoV_N2-F	
		2019-nCoV_N2-R	
		2019-nCoV_N2-P	
	N	2019-nCoV_N3-F	
		2019-nCoV_N3-R	
		2019-nCoV_N3-P	
	Human RNase P	RP-F	
		RP-R	
		RP-P	

**#**: Primer/Probe sequences not included; Adapted from Vogels *et al*.

There have been several published reports of laboratory-confirmed COVID-19 cases across the globe based on the RT-qPCR technology. However, it was observed that they also reported different indicators of positive test results. Also, instances of discharged COVID-19 patients who later tested positive for SARS-CoV-2 RNA have been documented [8]. These controversies, together with the earlier observation that several published studies reported the use of diverse reagents, protocols and analysis methods for qPCR assay [6], create doubts about the accuracy/reliability of the RT-qPCR for diagnosis of COVID-19. In this commentary, we reviewed published works on use of the RT-qPCR for diagnosis of COVID-19 in infected individuals and examined the possibility of standardization of the assay procedure to facilitate generation of globally comparable results. The varying Ct values reported in some of the relevant published works were critically appraised and discussed *vis-à-vis* challenges and prospects. Reports of varying indicators of positive/negative RT-qPCR test results for COVID-19 diagnosis: In performing the RT-qPCR, the indicator of detectable amplification of the viral RNA is graphically known as quantitation cycle [6], commonly reported as cycle threshold value (Ct) [5]. The Ct for a given clinical sample represents the amplification cycle that crosses the threshold line programmed by individual scientists using the assay in a specific brand of real-time thermal cycler. It was reported that Ct values of 25 to 28 were usually appropriate; when more than 28 cycles, detection of non-specifically precipitated sequences could occur or lead to variable results due to inactivation of Taq polymerase. A clinical sample being assayed must not only show amplification signal/cycle crossing the threshold line within cycles set for positivity, it must also have relatively low Ct value/number (Ct value being inversely related to copy numbers of template RNA/DNA in a given sample) to be considered positive compared to appropriate RT-qPCR controls. The findings of this review show that the Ct value varies with different sample types, RT-qPCR system used and brand or model of thermal cycler [7].

For the diagnosis of COVID-19, different Ct values have been used, ranging between 16.9 and 38.8 for various clinical samples. Although, Ct values < 40 is generally recommended as indicator of SARS-CoV-2 RNA positivity [7], some workers reported that samples with Ct values > 33.33 or 35, or ≥ 39.2 or 40 could be considered as negative [7]. Similarly, a verification study of commercial RT-qPCR tests of residual patient samples reported that strong positive samples typically have Ct values ranging from 15-24, and values of 25-30 for moderately positive samples. These differences in Ct cut-off values could be due to the types of clinical samples, sampling time and protocols employed by the various groups of workers. From the instances cited above, it implies that some clinical samples might be considered negative with Ct values set below 30, 33.33, 35 or 37 whereas such would be considered positive using cut-off values ≥ 40 for negative samples as recommended by the CDC. In a related study, it was reported that delayed transport or multiple freeze-thaw cycles of clinical samples should be discouraged as the Ct value may exceed 31 for such (i.e. approaching limit of viral RNA detection). The American Society for Microbiology also opined that if the limit of detection (LOD) of a given RT-qPCR kit is too high, SARS-CoV-2-infected patients might not test positive (i.e. high rate of false-negative results), whereas if the LOD is too low, contamination can become a major problem, as the kit would detect the tiniest amounts of viral RNA, leading to false-positive test results.

It is important to state that the various RT-qPCR protocols use different primer-probe sets targeting diverse segments of the SARS-CoV-2 genome [7,9]. These protocols may not have similar analytical or clinical sensitivity and specificity even when used for the same COVID-19 clinical sample. It must be borne in mind that analytical sensitivity and specificity are different from their corresponding clinical counterparts, and these parameters have not even been established for COVID-19 diagnosis [6]. It is also noteworthy that different RT-qPCR kits for SARS-CoV-2 detection have different reagents, and when the same, their concentrations in the reaction mixture may be different as seen with the assays/protocols developed by the US CDC, China CDC, Charité-Universitätsmedizin Berlin, Germany and Hong Kong University [5,9,10]. As observed by Vogels et al. [7] who critically compared the analytical efficiencies and sensitivities of these four RT-qPCR assays, each of them is likely to have different sensitivity/specificity, and possibly different accuracies. They concluded that all the primer-probe sets for these four assays (developed by different scientists and listed by the WHO) can be used to detect SARS-CoV-2 as long as the limitations of each assay are recognized. However, they noted that the different assays have clear variations in their abilities to differentiate between true negatives and positives when low titer of SARS-CoV-2 is present in a given sample. This becomes crucial when samples from asymptomatic COVID-19 suspects are tested, in which case, the CoV RNA may be quite low to indicate early viral replication.

Although the RT-qPCR detection of SARS-CoV-2 RNA does not necessarily mean viable virus is present in the samples, appropriate interpretation of the diagnostic test results is guided by the clinical manifestations and epidemiologic history of the patient. A positive laboratory result therefore means the tested person has active SARS-CoV-2 infection (even if clinical signs/symptoms are absent) and hence, considered infectious to susceptible human contacts [3]. Conversely, two consecutive negative test results indicate that the individual has no detectable SARS-CoV-2 RNA as at the time of sampling and is considered not infected or infectious. However, when history of the patient points to probable exposure to the CoV, self-isolation for a period of 14 days is recommended for manifestation of pertinent signs and symptoms, otherwise, the patient is discharged as uninfected [8]. It has however been suggested that negative results do not exclude SARS-CoV-2 infection [3]. Any positive/infected person in isolation facility undergoing clinical management can only be discharged as having recovered from COVID-19 when RT-qPCR returns negative results twice from two or more clinical samples collected, at least, 24 hours apart [4,8]. This further highlights the importance of the assay. With respect to sample types, we observed in this review that different clinical samples displayed different sensitivities for SARS-CoV-2 detection. For instance, samples from the lower respiratory tract of COVID-19 patients showed greater sensitivity. It is generally believed that good sample collection is a prerequisite for accurate laboratory diagnosis, but scientists are not yet sure what “good specimen collection” means for COVID-19. Although the US CDC recommends nasopharyngeal swabs for COVID-19 diagnosis, the issue of sampling is still evolving.

Considering the pathogenicity of SARS-CoV-2 and the sensitive nature of the current pandemic, and in order to ensure assay reliability, the China and US CDCs designed different protocols for their respective RT-qPCR kit. For the US CDC, as shown in Table 1, detection of SARS-CoV-2 RNA requires four RT-qPCRs (that detect the SARS-CoV-2 N1, N2 and N3 genes, and human RNase P gene) for each COVID-19 clinical sample. However, many centers and referral laboratories, guided by the US CDC, require three RT-qPCRs (that detect N1, N2 and RNase P genes) per sample. Nevertheless, diagnostic laboratories all over the world are caught between which of the existing RT-qPCR assays to adopt [7]. Other factors that may impact accuracy/reliability of RT-qPCR include human errors in sample processing/preparation and testing procedure, presence of RT and PCR inhibitors in reaction mixture, and contamination of sample by RNase. These can be overcome by ensuring that sampling and sample transport are done by trained healthcare workers, assays are conducted by personnel with demonstrable expertise and experience in RT-qPCR, and inclusion of RNase inhibitor in the assay. As an RNA virus with high propensity for mutational changes in the genome, evolution of SARS-CoV-2 with the emergence of strains having diverse replication efficiencies and clinicopathological manifestations over the course of the pandemic, could be another reason accounting for differences in analytical sensitivity of available RT-qPCR assays and consequently, the divergent test results obtained. So far, different strains of the virus have been identified. Although at the moment, several RT-qPCR assays for COVID-19 diagnosis are available, none is perfect yet. It is advocated that positive-negative Ct cut-off values should be locally validated when establishing RT-qPCR assays for SARS-CoV-2 detection [7].
